# Supplementary material for: Kdm4a is an activity downregulated barrier to generate engrams for memory separation
Source: Nat Commun. 2024 Jul 13;15:5887. doi: 10.1038/s41467-024-50218-y (PMC11246488; doi:10.1038/s41467-024-50218-y)

Kdm4a is an activity downregulated barrier to generate new engrams for memory separation

Authors:
Xiuxian Guo[1], Pengfei Hong[1], Songhai Xiong[1], Yuze Yan[1], Hong Xie[3*], Ji-Song Guan[1,2*]

Affiliations:
[1]School of Life Science and Technology, ShanghaiTech University, Shanghai, China
[2]State Key Laboratory of Advanced Medical Materials and Devices, ShanghaiTech University, Shanghai, China
[3]Institute of Photonic Chips, University of Shanghai for Science and Technology, Shanghai, China

*Correspondence:
Ji-Song Guan guanjs@shanghaitech.edu.cn;
Hong Xie hongxie@usst.edu.cn

Supplementary Figure 1-16

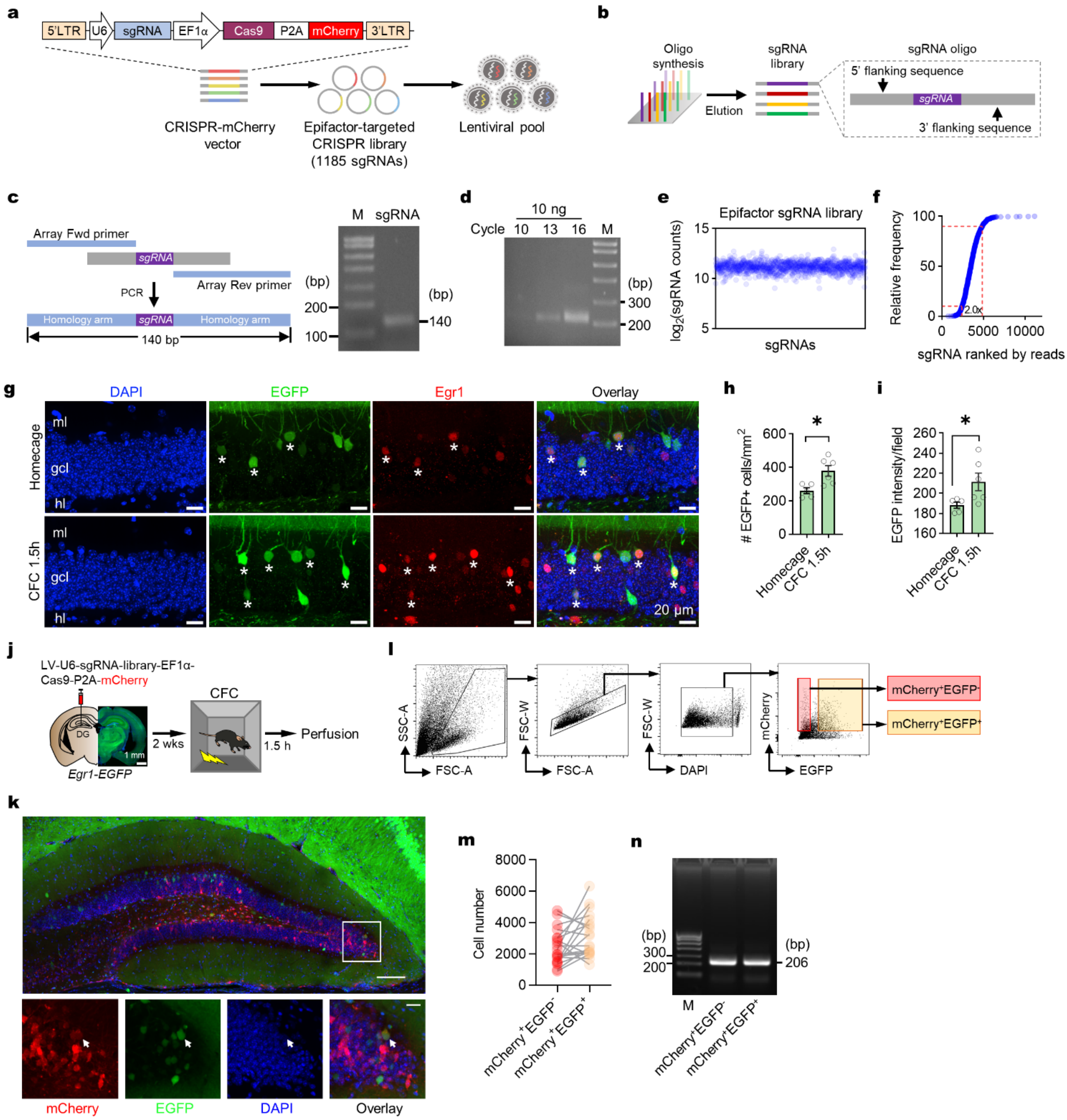

Supplementary Figure 1

Supplementary Figure 1. Construction of in vivo CRISPR-Cas9 screening library.

a, sgRNA oligos were designed to insert into plentiCRISPRv2-mCherry vector.

b, The synthesized sgRNA oligo contains sgRNA sequence and two flanking sequences.

c, Homology arms are introduced on both sides of the sgRNA library oligonucleotide fragment through PCR amplification for homologous recombination.

d, The sgRNA fragments on the sgRNA library plasmids were amplified by PCR and then used for electrophoresis. PCR products within the exponential amplification (cycle number cycle=13) were selected for next-generation sequencing-based quality control.

e, Quality control results of sgRNA library plasmids. The sgRNAs read count is distributed heterogeneously across the target genes.

f, Frequency distribution analysis of each sgRNA reads. the ratio of the number of reads at the 90th percentile and the 10th percentile is 2.01, indicating that the sgRNA library plasmid has high homogeneity.

g, Representative images of Egr1-EGFP-expressing DG GCs after CFC.

h, Quantification of EGFP positive cell number in homecage or CFC. Homecage, n = 5; CFC, n = 6. Two-tailed unpaired t-test, t_9 = 3.077, *p* = 0.0132. Data are presented as mean ± s.e.m.

i, Quantification of EGFP fluorescence intensity in homecage or CFC. Homecage, n = 5; CFC, n = 6. Two-tailed unpaired t-test, t_9 = 2.301, *p* = 0.0469. Data are presented as mean ± s.e.m.

j, Stereotactic injection of lentiviral sgRNA library into the DG of *Egr1-EGFP* mice. Mice were perfused 1.5 h after contextual fear conditioning and fixed for immunohistochemistry (IHC).

k, Immunofluorescence staining of DG cells infected with lentiviral sgRNA library. $mCherry^+$ cells are neurons infected with the lentiviral sgRNA library. $EGFP^+$ is CFC-activated $Egr1^+$ neurons. Scale bar, 250 μm.

l, Gating strategy for *in vivo* CRISPR screen in *Egr1-EGFP* mouse DG.

m, The numbers of two groups of mouse $mCherry^+EGFP^-$ and $mCherry^+EGFP^+$ cells collected by FACS. n=20.

n, PCR amplification of the sgRNA fragment (206 bp) from the integrated genomic DNA of $mCherry^+EGFP^-$ and $mCherry^+EGFP^+$ cells. Purified PCR products were used for next-generation sequencing.

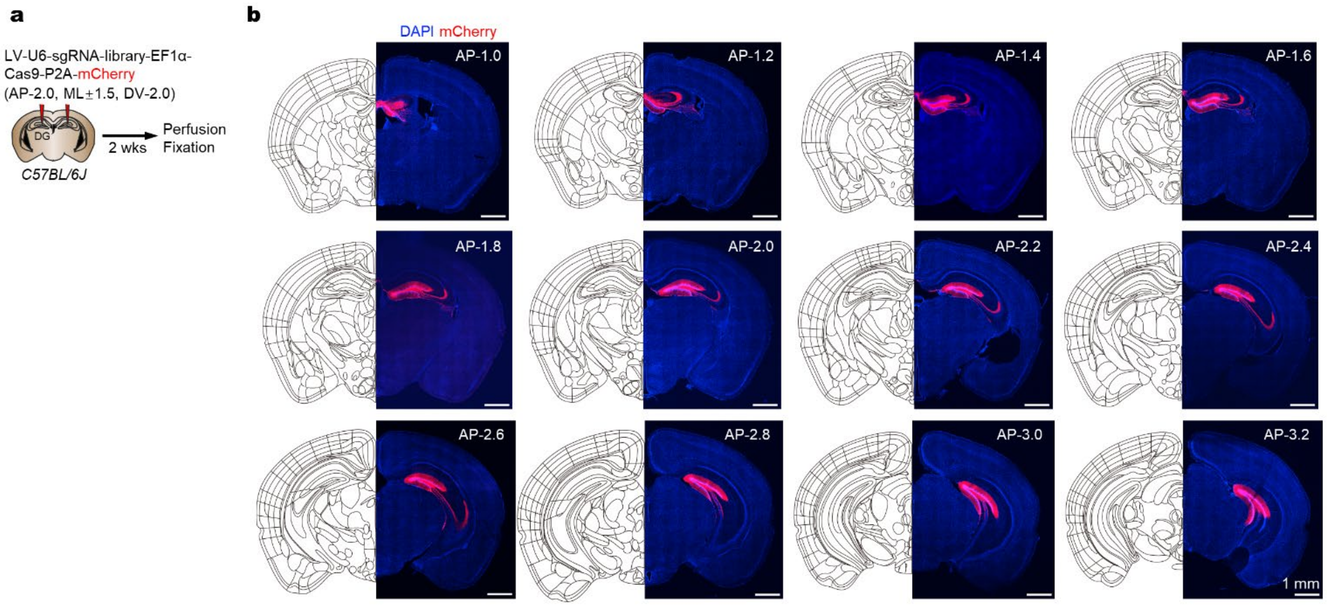

Supplementary Figure 2

Supplementary Figure 2. Characterization of lentiviral spread in the DG.

a, Schematics of delivery of lentivirus into the DG.

b, Representative images of serial sections of LV-injected mouse brain.

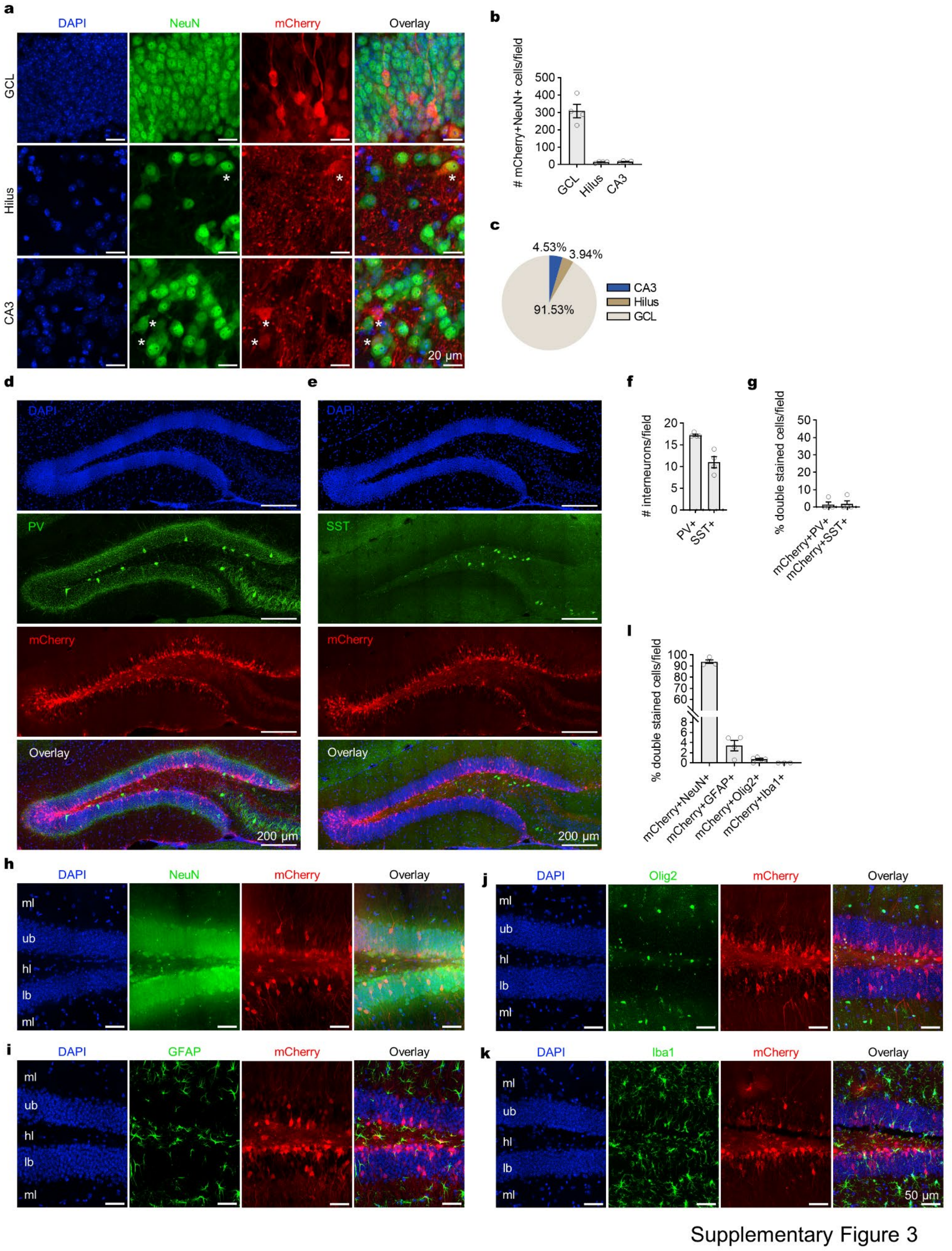

Supplementary Figure 3. Estimation of multiplicity of lentiviral infection in the DG.

a, Representative images of co-staining of mCherry and NeuN in the GCL, Hilus and CA3.

b, The number of mCherry+NeuN+ double positive cells in the GCL, Hilus and CA3. **c**, The percentage of mCherry+NeuN+ double positive cells in the GCL, Hilus and CA3. **d**, Representative images of co-staining

of mCherry and PV+ INs.

e, Representative images of co-staining of mCherry and SST+ INs.

f, The number of INs in the DG.

g, The percentage of double stained cells of mCherry and different interneuron markers. **h**, Representative images of co-staining of mCherry and NeuN.

i, Representative images of co-staining of mCherry and GFAP.

j, Representative images of co-staining of mCherry and Olig2.

k, Representative images of co-staining of mCherry and Iba1.

l, The percentage of double stained cells of mCherry and different cell markers.

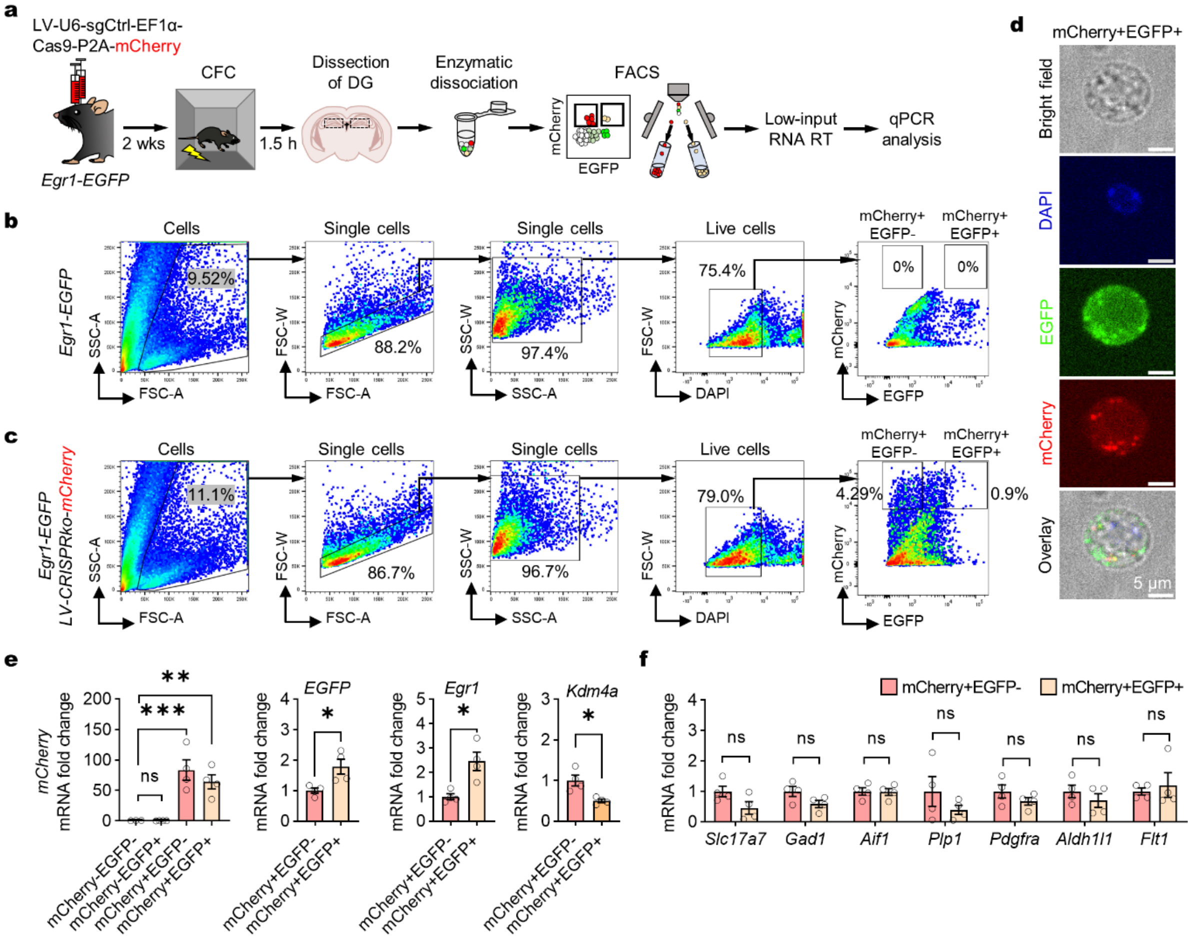

Supplementary Figure 4

Supplementary Figure 4. Analysis of the cell type specificity of neurons isolated from Egr1-EGFP-based cell sorting.

a, Schematics of the isolation of lentiviral infected cells from the DG.

b-c, Representative FACS plots showing the gating strategy for the identification of mCherry-expressing cells in the DG.

d, Representative images of FACS sorted mCherry+EGFP+ cell.

e, Expression levels of *mCherry*, *EGFP*, *Egr1* and *Kdm4a* mRNA in the FACS sorted cells. *mCherry*: One-way ANOVA followed by Dunnett test, $F_{3,\ 11}$ = 14.99, p = 0.0003. ** p = 0.0069, *** p = 0.0010. Two-tailed unpaired t-test, n = 4, *EGFP*: t_6 = 3.040, * p = 0.0228; *Egr1*: t_6 = 3.663, * p = 0.0105; *Kdm4a*: t_6 = 3.576, * p = 0.0117. Data are presented as mean ± s.e.m.

f, Cell-type specific gene expression in the FACS sorted cells. Two-tailed unpaired t-test, n = 4. Data are presented as mean ± s.e.m.

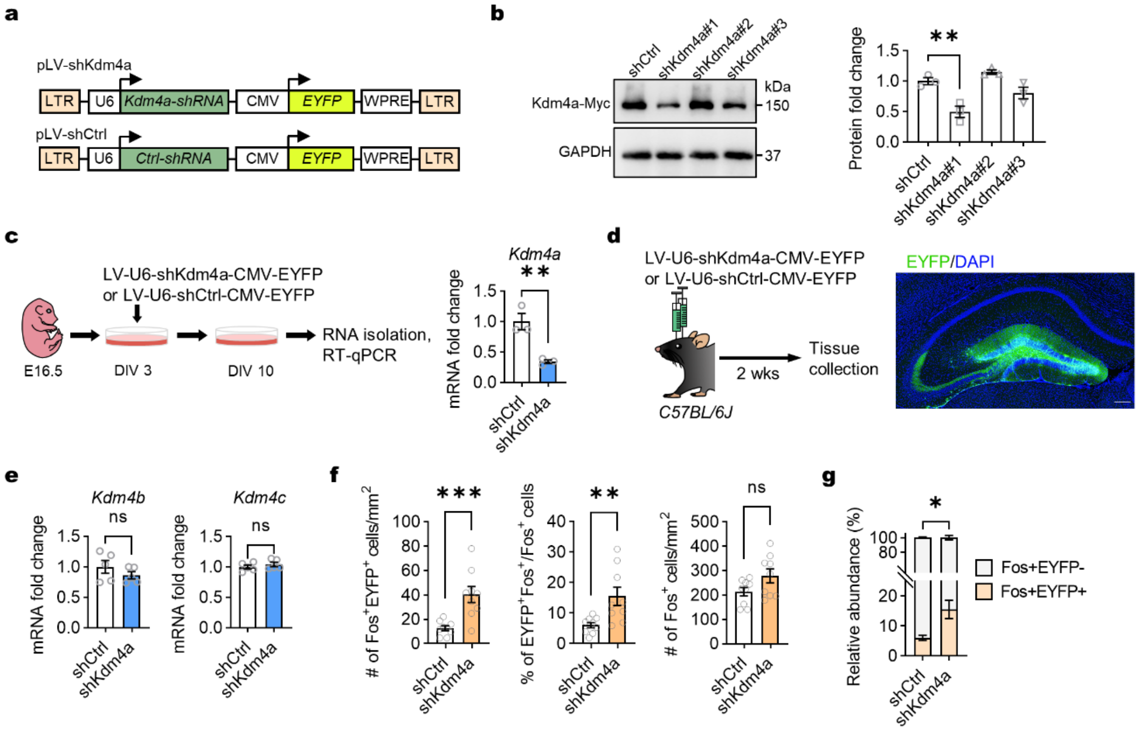

Supplementary Figure 5. Validation of Kdm4a knockdown efficiency.

a, Illustration of the RNAi lentiviral vector (shKdm4a) and control vector (shCtrl).

b, Western blot detection of Kdm4a shRNA knockdown efficiency. shCtrl n = 3, shKdm4a n = 3. One-way ANOVA followed by Dunnett test, $F_{3,8} = 14.51$, $p = 0.0013$. ** $p = 0.004$. Data are presented as mean ± s.e.m.

c, RT-qPCR detection of Kdm4a shRNA knockdown efficiency in cultured neurons (DIV10). shCtrl n = 3, shKdm4a n = 3. Two-tailed unpaired t-test, $t_4 = 4.867$, ** $p = 0.0082$. Data are presented as mean ± s.e.m.

d, The shKdm4a or shCtrl lentivirus was injected stereotaxically into the DG of *C57BL/6J* mouse. After 2 weeks of infection, mouse brain was either perfused for observation of lentiviral expression, or dissected and collected for biochemical analysis. Scale bar = 250 μm.

e, shKdm4a lentivirus has no effect on the expression of other Kdm4 family genes (*Kdm4b*, *Kdm4c*) in the DG. shCtrl, n = 5; shKdm4a, n = 5. Two-tailed unpaired t-test, *Kdm4b*: $t_8 = 1.174$, $p = 0.274$; *Kdm4c*: $t_8 = 0.9256$, $p = 0.3817$. Data are presented as mean ± s.e.m.

f, (Left) The number of Fos^+EYFP^+ double-positive cells in shKdm4a mice is significantly more than that in the shCtrl mice. shCtrl, slices = 9; shKdm4a, slices = 9. Two-tailed unpaired t-test, $t_{16} = 4.027$, *** $p = 0.001$. (Middle) The percentage of Fos^+EGFP^+ cells/Fos^+ cells in the shKdm4a group was significantly higher than that in the shCtrl group. Two-tailed unpaired t-test, $t_{16} = 3.019$, ** $p = 0.0081$. (Right) The total number of Fos^+ cells in the shKdm4a group did not change significantly compared with the shCtrl group. Two-tailed unpaired t-test, $t_{16} = 1.95$, $p = 0.0689$. Data are presented as mean ± s.e.m.

g, The shKdm4a mice showed an increase in the fraction of Fos^+EYFP^+ cells and a reduction in the fraction of Fos^+EYFP^- cells. shCtrl n = 9, shKdm4a n = 9. One-way ANOVA followed by Bonferroni test, $F_{3,32} = 425.6$, $p < 0.0001$. * $p = 0.0297$. Data are presented as mean ± s.e.m.

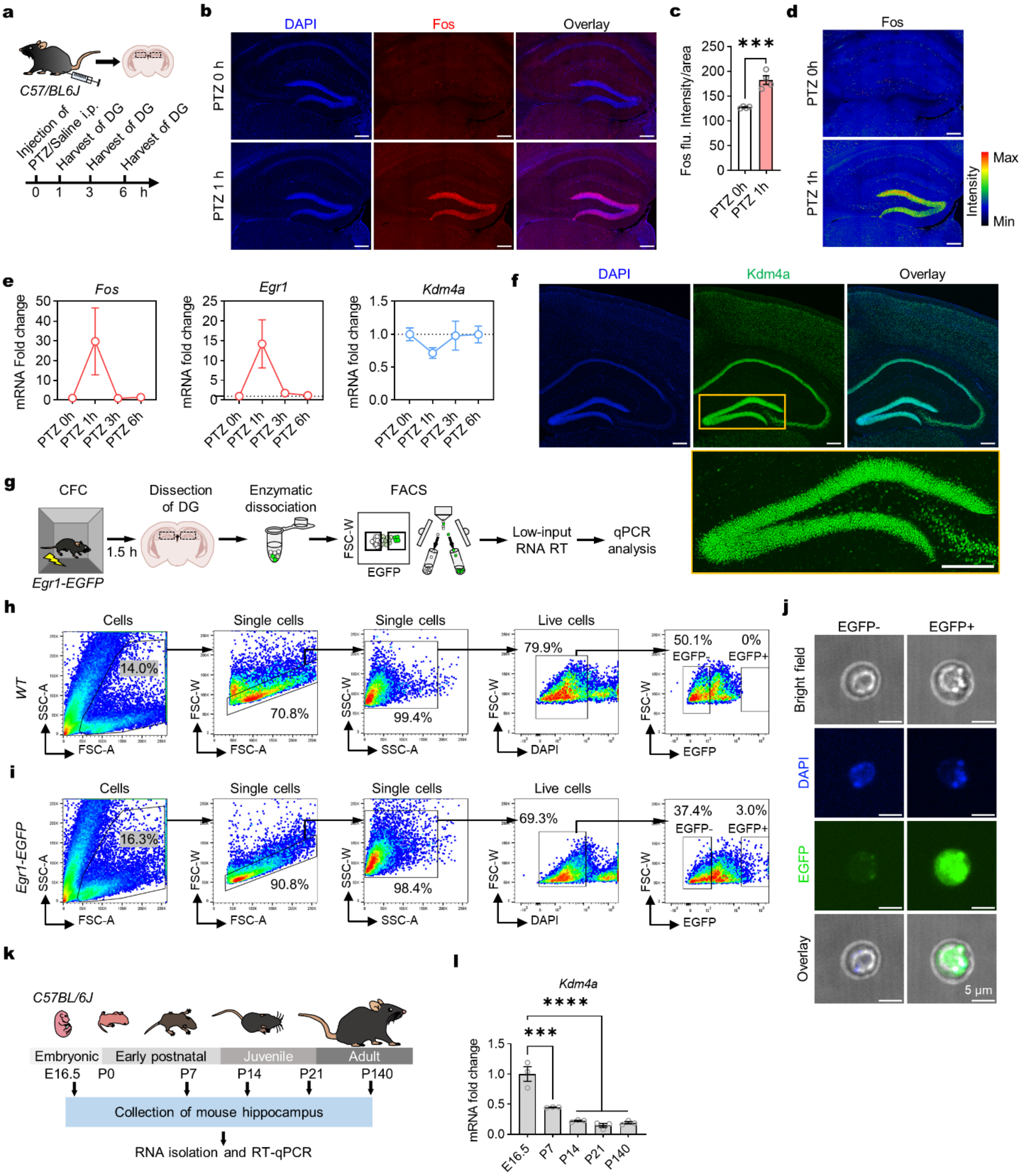

Supplementary Figure 6

Supplementary Figure 6. Kdm4a is an activity-downregulated gene in the DG GCs.

a, Diagram of in vivo activation of DG GCs using PTZ. After intraperitoneal injection of 50 mg/kg Pentylenetetrazole (PTZ) or saline in C57BL/6J mice for 1, 3 and 6 h, the DG was dissected and RNA was extracted for RT-qPCR detection.

b, Fos staining results of DG granule cells activated by injection of PTZ. Scale bar, 250 μm.

c, Quantitative results of Fos signal in the DG granule cell layer. PTZ 0 h, slices = 4; PTZ 1 h, slices = 4. Two-tailed unpaired t-test, t_6 = 6.34, *** p = 0.0007. Data are presented as mean ± s.e.m.

d, Heat map of hippocampal Fos immunofluorescence signal intensity. PTZ specifically activates hippocampal DG GCs. Scale bar, 250 μm.

e, The expression of IEGs including *Fos* and *Egr1* increased with the activation of DG neurons (1 h), and

then decreased to the basal level. Conversely, *Kdm4a* expression decreased (1 h) with activation of DG neurons and then returned to basal levels. n = 3 each group.

f, Representative images of Kdm4a protein levels in the adult mouse hippocampus. Scale bar, 250 μm.

g, Schematics of the isolation of the CFC-activated EGFP+ neurons from the DG of Egr1-EGFP transgenic mice.

h-i, Representative FACS plots showing the gating strategy for the identification of EGFP-expressing cells in the DG.

j, Representative images of FACS sorted EGFP- cell and EGFP+ cell.

k, The hippocampal tissue was collected at 5 time points during mouse development (E16.5, P7, P14, P21 and P140) for RNA extraction and RT-qPCR detection.

l, The mRNA level of *Kdm4a* in the hippocampus is relatively high during the embryonic stage and decreases significantly in the early postnatal stage (P7). Kdm4a transcription levels do not change significantly from youth to adult (P14-P140). n = 3 per group. Ordinary One-way ANOVA is used, $F_{4,10} = 37.72$, $p < 0.0001$. *** $p = 0.0002$, **** $p < 0.0001$. Data are presented as mean ± s.e.m.

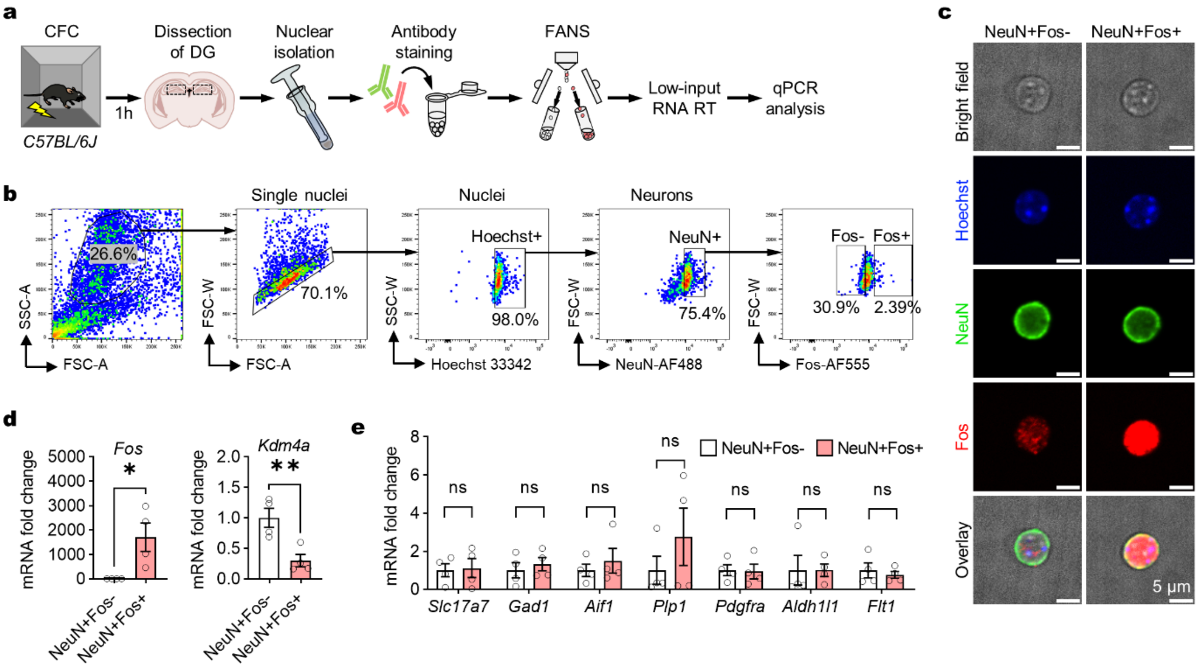

Supplementary Figure 7

Supplementary Figure 7. Detection of memory-associated Kdm4a levels with fluorescence-activated nuclei sorting.

a, Schematics of flow sorting strategy for the isolation of fear memory-associated nuclei from the DG.

b, Representative FANS plots showing the gating strategy for the identification of NeuN+Fos+ nuclei in the DG.

c, Representative images of FANS sorted nuclei.

d, Fos (left) and Kdm4a (right) mRNA expression levels in the NeuN+Fos- or NeuN+Fos+ nuclei. Two-tailed unpaired t-test, n = 4, *Fos*: t_6 = 2.949, * p = 0.0256; *Kdm4a*: t_6 = 3.732, ** p = 0.097. Data are presented as mean ± s.e.m.

e, Several maker gene mRNA expression levels in the NeuN+Fos- or NeuN+Fos+ nuclei. Two-tailed unpaired t-test, n = 4. Data are presented as mean ± s.e.m.

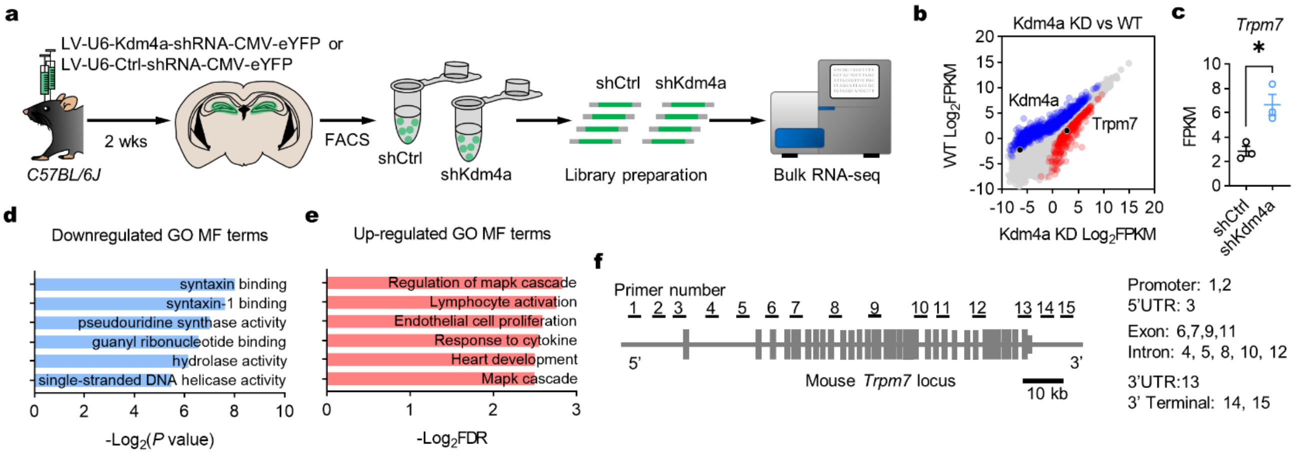

Supplementary Figure 8

Supplementary Figure 8. Transcriptomic analysis of Kdm4a knockdown in the DG GCs.

a, Transcriptome analysis of Kdm4a knockdown in DG GCs. The shKdm4a or shCtrl lentivirus was injected stereotaxically into the DG of *C57BL/6J* mouse. DG was dissected 2 weeks later and dissociated into single cells for cell sorting. Dentate gyrus EYFP$^+$ cells infected with shKdm4a or shCtrl virus were sorted and collected separately, and then subjected to Smart-seq. n = 3, cells = 2000 each replicate.

b, The differentially expressed genes (DEGs) between shCtrl and shKdm4a. Red: upregulated genes in shKdm4a group; blue: downregulated genes in shKdm4a group.

c, The FPKM value of *Trpm7* is increased in Kdm4a-knockdown cells. shKdm4a n=3, shCtrl n=3. Two-tailed unpaired t-test, t_4 = 4.032, * p = 0.0157. Data are presented as mean ± s.e.m.

d, Gene ontology analysis of the downregulated DEGs.

e, Gene ontology analysis of the upregulated DEGs..

f, The ChIP-qPCR primers were designed to target on the sequence of mouse *Trpm7* gene loci. The locations targeted by the primers include the promoter, 5' UTR, exons, introns, 3' UTR and 3' terminal regions on the *Trpm7* gene loci.

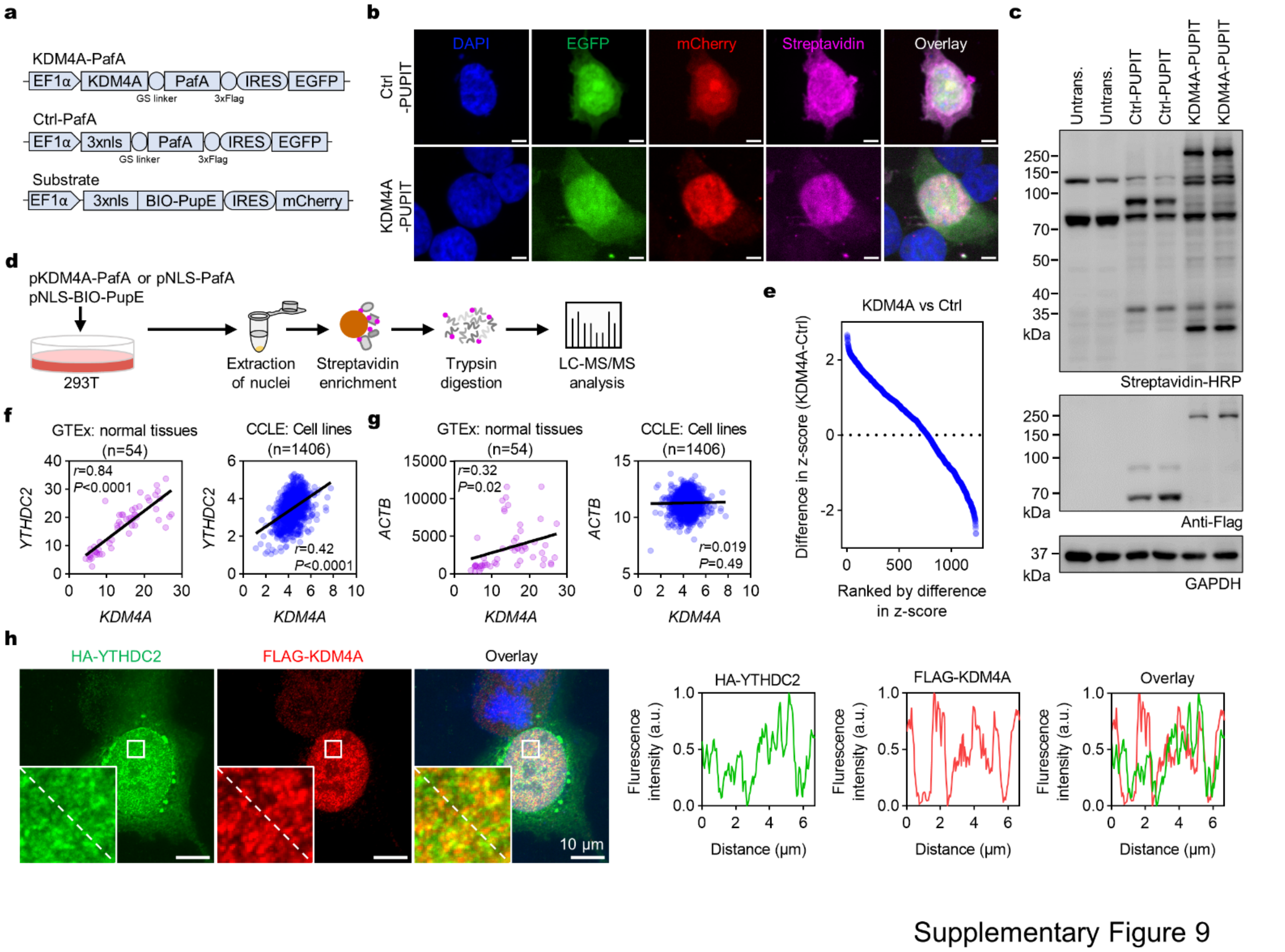

Supplementary Figure 9. Identification of the Kdm4a proximal proteome using PUP-IT labeling.

a, Design of the KDM4A PUP-IT experiment. Three PUP-IT plasmids: pCMV-3xFlag-KDM4A-PafA-IRES-EGFP (KDM4A-PafA); pCMV-3xNLS-3xFlag-PafA-IRES-EGFP (Ctrl-PafA); pCMV-3xNLS-BIO-PupE -IRES-mCherry (NLS-BIO-PupE).

b, Subcellular localization of the KDM4A PUP-IT system. KDM4A-PafA or Ctrl-PafA and NLS-BIO-PupE were co-transfected into 293T cells. KDM4A is a nuclear protein, and the fusion protein KDM4A-PafA distribute in the nucleus. Ctrl-PafA is fused with the nuclear localization sequence (3xNLS) and also distribute in the nucleus. Scale bar, 5 µm.

c, Western blot detection of KDM4A-PUPIT labeled nuclear proteins. After 24 h of transfection, cells were lysed and nuclear proteins were enriched for WB detection. Compared with the untransfected group (Untrans.) and the control group (Ctrl-PUPIT), the KDM4A-PUPIT system significantly labeled the KDM4A-associated proteins.

d, Diagram of KDM4A PUP-IT proximity labelling experiments.

e, MS analysis of the KDM4A-associated proteins that were enriched by PUP-IT.

f, Correlation of KDM4A with YTHDC2 in expression in normal tissues (left) and cell lines (right), based on data from Genotype Tissue Expression (GTEx) and Cancer Cell Line Encyclopedia (CCLE), respectively.

g, Correlation of KDM4A with ACTB in expression in normal tissues (left) and cell lines (right).

h, Co-localization of Flag-KDM4A and HA-YTHDC2 in U2-OS cell. Scale bar, 10 µm.

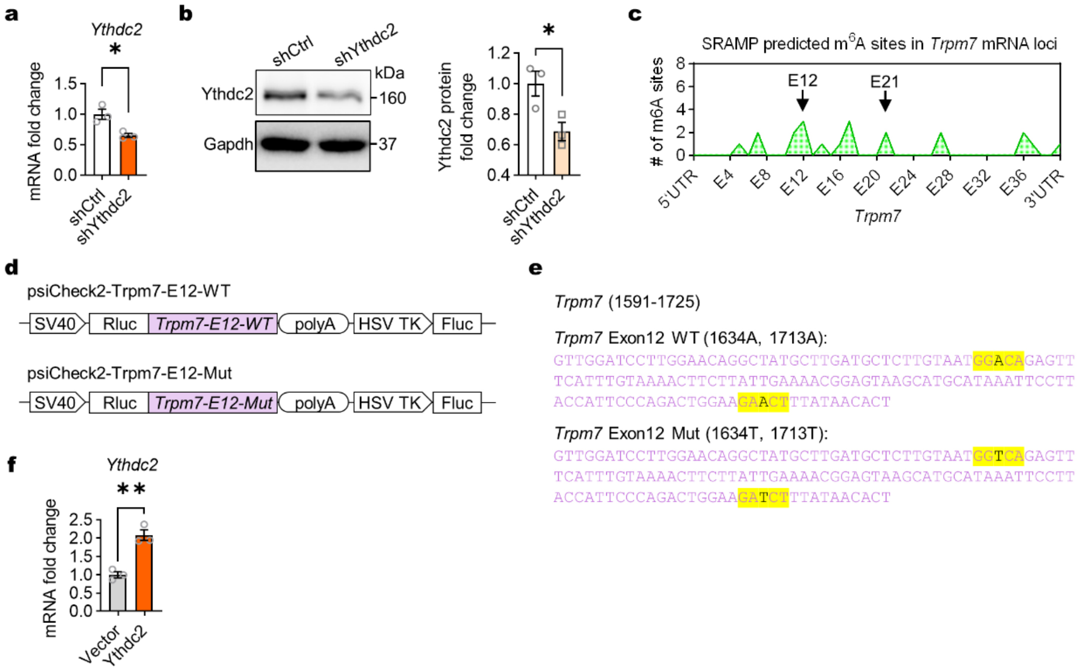

Supplementary Figure 10

Supplementary Figure 10. Ythdc2 stabilizes *Trpm7* mRNA through binding to m^{6}A sites.

a, RT-qPCR detection of Ythdc2 knockdown efficiency. shCtrl, n = 3, shYthdc2, n = 3. Two-tailed unpaired t-test, t_4 = 3.829, * p = 0.0186. Data are presented as mean ± s.e.m.

b, Western blot detection of Ythdc2 knockdown efficiency. shCtrl, n = 3, shYthdc2, n = 3. Two-tailed unpaired t-test, t_4 = 3.098, * p = 0.0363. Data are presented as mean ± s.e.m.

c, SRAMP software predicts the m6A site located on mouse *Trpm7* mRNA. Arrows indicate Exon 12 and Exon 21 respectively.

d, Construction of dual-luciferase reporter system plasmid. The *Trpm7* mRNA Exon 12 WT or Mut fragments were inserted into the 3'UTR region of Renilla luciferase (Rluc) respectively.

e, The Exon12 sequence of *Trpm7* and the m^{6}A sites above it (the yellow area is the 2 nucleotides on both sides of the m^{6}A site, and the black letters represent the m^{6}A site). The m^{6}A site was replaced by T to disrupt the recognition of m^{6}A binding proteins.

f, RT-qPCR detection of Ythdc2 CDS overexpression efficiency. Vector, n = 3, Ythdc2-Myc, n = 3. Two-tailed unpaired t-test, t_4 = 6.438, ** p = 0.003. Data are presented as mean ± s.e.m.

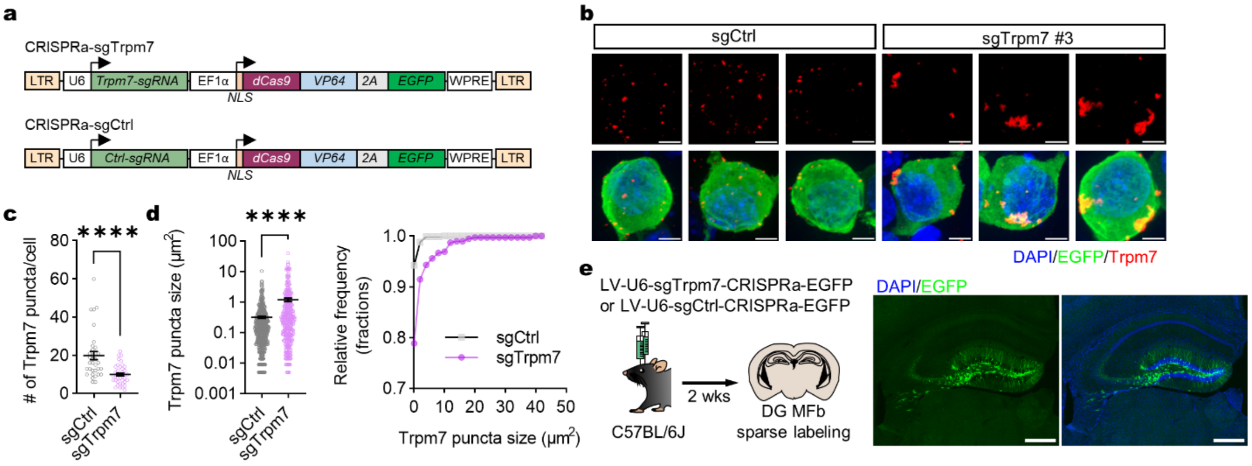

Supplementary Figure 11

Supplementary Figure 11. CRISPR activation of *Trpm7* expression increased the size of Trpm7 puncta on the plasma membrane.

a, CRISPRa-based transcription activation system. Construction of LV-CRISPRa-EGFP-sgTrpm7/sgCtrl plasmid.

b, Immunofluorescence staining of Trpm7 on the cell membrane. The plasmid LV-CRISPRa-EGFP-sgTrpm7#3 and the control plasmids were transfected into the N2a cell line respectively. After 48 h of transfection, cells were fixed for TRPM7 immunofluorescence staining. Scale bar,2 μm.

c, Transcriptional activation of *Trpm7* in N2a cells reduced the number of Trpm7 puncta on the membrane. sgCtrl, cells = 34; sgTrpm7, cells = 39. Two-tailed unpaired t-test, t_{71} = 4.509, **** $p < 0.0001$. Data are presented as mean ± s.e.m.

d, (Left) Transcriptional activation of Trpm7 significantly increased the size of Trpm7 puncta on the membrane. sgCtrl, puncta = 674; sgTrpm7, puncta = 388. Two-tailed unpaired t-test, t_{1060} = 6.78, **** $p < 0.0001$. Data are presented as mean ± s.e.m. (Right) Frequency distribution of Trpm7 puncta area on the cell membrane.

e, The CRISPRa-sgTrpm7 or sgCtrl lentivirus was injected into the DG of C57BL/6J mice for sparse labeling of MFBs. Scale bar, 250 μm.

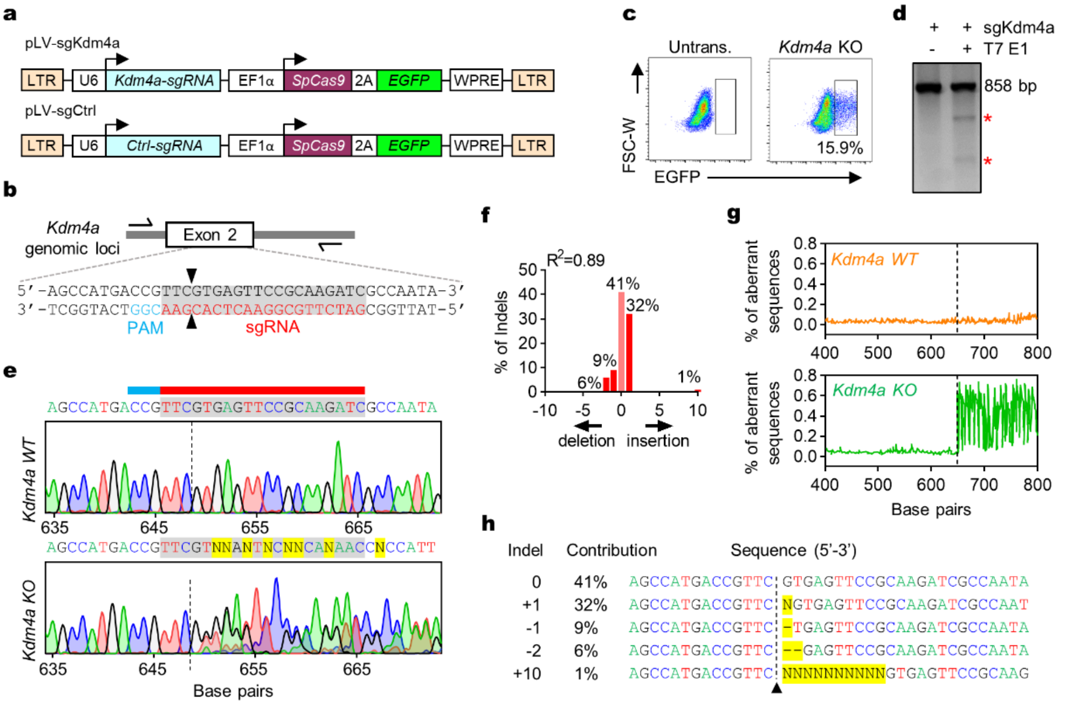

Supplementary Figure 12

Supplementary Figure 12. Validation of knockout efficiency of Kdm4a sgRNA.

a, sgRNA targeted at Kdm4a was inserted into the pLenti-CRISPRv2-EGFP lentiviral vector.

b, Design of sgRNA targeting mouse *Kdm4a* Exon 2. Red letter is sgRNA sequence, blue letter is the PAM (protospacer adjacent motif) motif, and the black triangle indicates the cleavage site of SpCas9 endonuclease. Primers were used to amplify DNA fragments containing the Kdm4a sgRNA sequence.

c, Cell sorting of N2a cells transfected with sgKdm4a plasmid (EGFP$^+$ cells).

d, Results of T7 endonuclease 1 mismatch detection assay. Primers were designed to amplify an 858-nt-long gDNA fragment containing Kdm4a sgRNA targeting site. Adding T7 endonuclease 1 to the denatured PCR products will cut the mismatched DNA fragments. Red asterisks indicate the cut fragments.

e, Sanger sequencing analysis of gDNA of cells transfected with sgKdm4a versus sgCtrl. The genomic DNA containing the sgRNA targeting sequence was amplified by PCR. Purified PCR products were used for Sanger sequencing.

f, Using the ICE algorithm to analyze the Indel induction efficiency of Kdm4a sgRNA.

g, The proportion of abnormal sequences near the Cas9 cleavage site increases significantly in sgKdm4a cells when compared to sgCtrl cells.

h, Type and proportion of Indels introduced by Kdm4a sgRNA. The prominent mutation type is +1 Insertion (32%), causing a frameshift mutation in the mouse *Kdm4a* gene.

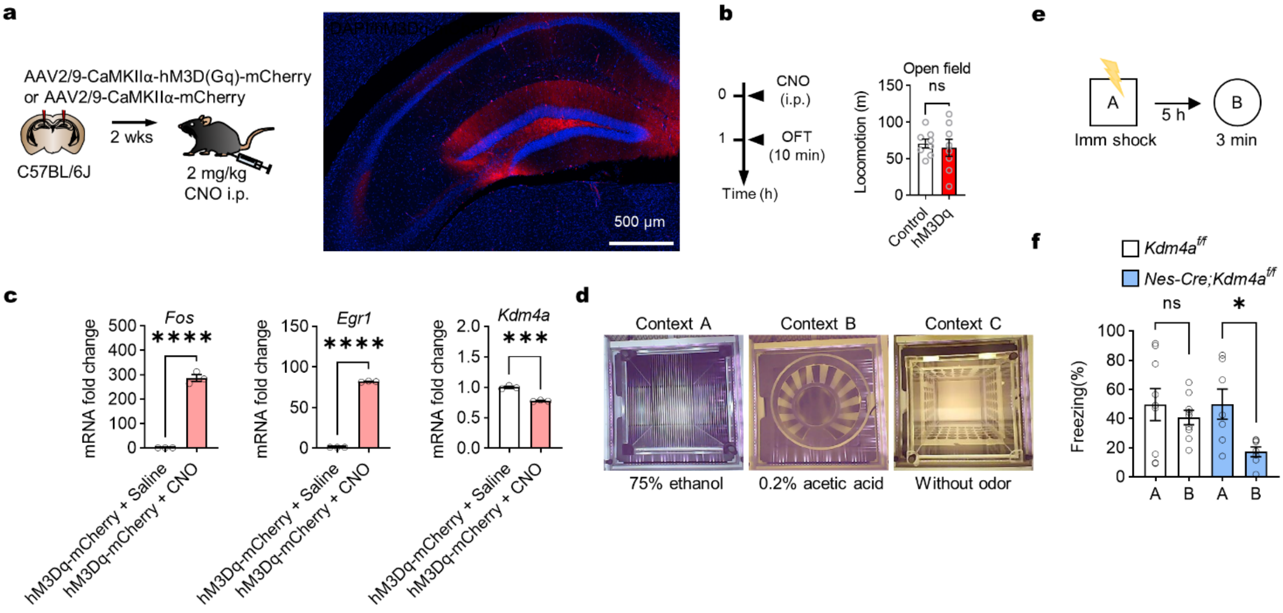

Supplementary Figure 13

Supplementary Figure 13. Chemogenetic activation of DG GCs in vivo.

a, Stereotaxic injection of AAV-hM3Dq-mCherry or control virus into the DG. Mice were then subjected 2 mg/kg CNO i.p. to activated DG GCs *in vivo*. Scale bar, 250 μm.

b, Open field test results. Activation of DG GCs did not increase the locomotor activity in mice. hM3Dq+CNO, n = 8; hM3Dq+Saline, n = 8. Two-tailed unpaired t-test, t_{14} = 0.4567, p = 0.6549. Data are presented as mean ± s.e.m.

c, *Fos* (left), *Egr1* (middle) and *Kdm4a* (right) mRNA expression levels in the chemogenetic activated DG. Two-tailed unpaired t-test, n = 3, *Fos*: t_4 = 20.19, **** $p < 0.0001$; *Egr1*: t_4 = 12.50, *** p = 0.0002. Data are presented as mean ± s.e.m.

d, The contexts used in the memory allocation test.

e, Schematics of a task for exploration of two different contexts close in time.

f, Kdm4a cKO mice exhibit lower freezing levels in B compared to A at 5 hours after immediate shock. Two-tailed unpaired t-test, *Kdm4a*$^{f/f}$, n = 9, t_{16} = 0.7349, ns p = 0.4730; *Nes-Cre;Kdm4a*$^{f/f}$, n = 7, t_{12} = 3.006, * p = 0.0110. Data are presented as mean ± s.e.m.

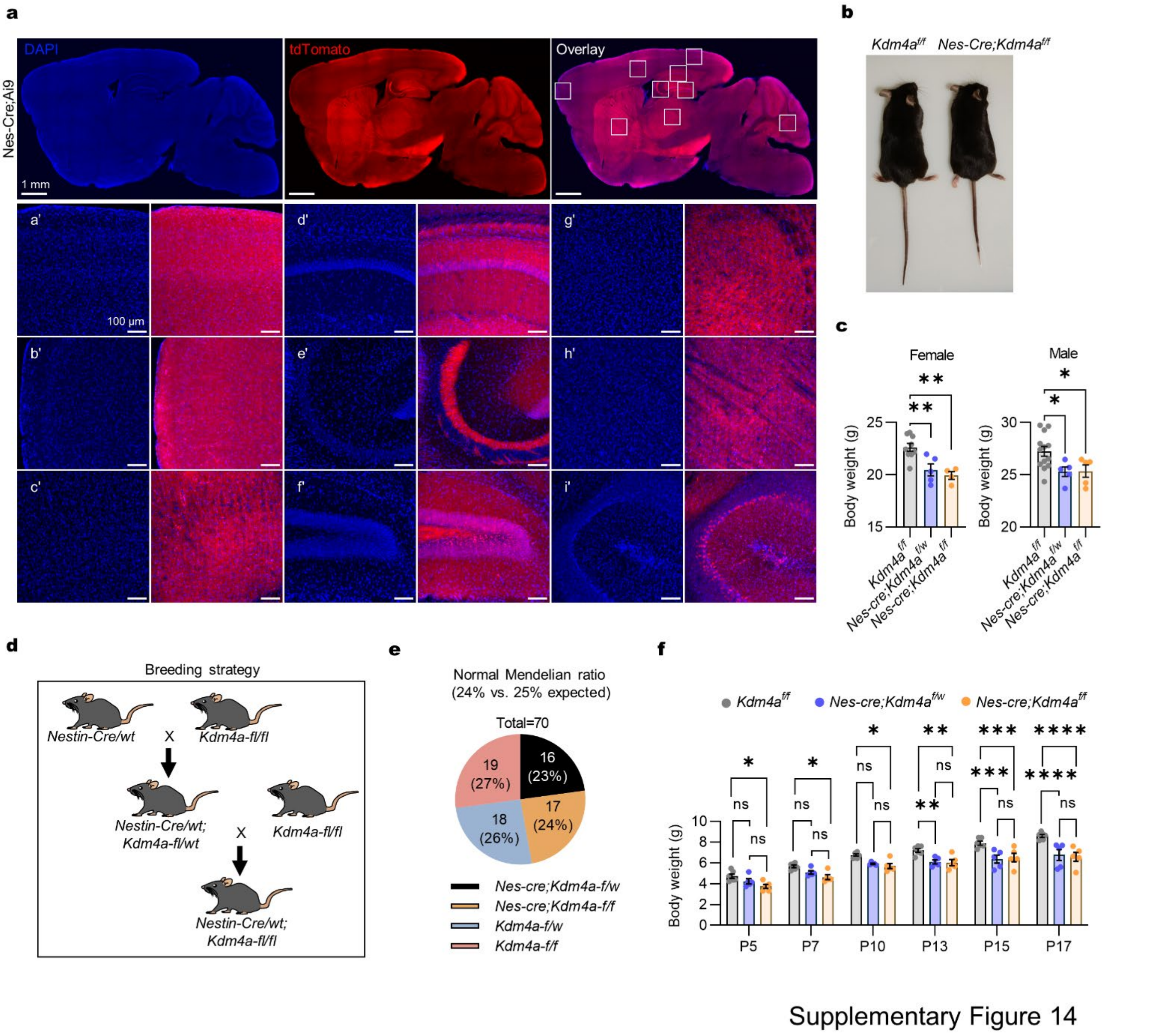

Supplementary Figure 14. The Kdm4a-cKO pups and mature mice displayed lower body weight.

a, Mapping of Nestin-dependent Cre expression using CAG-tdTomato (Ai9) reporter mice. Scale bar, 1mm.

b, 4-month-old male *Kdm4a*$^{f/f}$ and *Nes-Cre;Kdm4a*$^{f/f}$ mouse.

c, (Left) The body weight of P56 female *Kdm4a cKO* mice is significantly lower than control mice. *Kdm4a*$^{f/f}$, n = 9; *Nes-Cre;Kdm4a*$^{f/w}$, n = 5; *Nes-Cre;Kdm4a*$^{f/f}$, n = 4. One-way ANOVA followed by Sidak test, $F_{2,15}$ = 11.08, p = 0.0011. ** p < 0.01. (Right) The body weight of P56 male *Kdm4a cKO* mice is significantly lower than control mice. *Kdm4a*$^{f/f}$, n = 13; *Nes-Cre;Kdm4a*$^{f/w}$, n = 5; *Nes-Cre;Kdm4a*$^{f/f}$, n = 5. One-way ANOVA followed by Sidak test, $F_{2,20}$ = 4.784, p = 0.0201. * p < 0.05. Data are presented as mean ± s.e.m.

d, Breeding strategy for *Kdm4a cKO* mice.

e, The live *Nes-Cre;Kdm4a*$^{f/f}$ pups were born in numbers consistent with expected Mendelian ratio.

f, The body weight of *Kdm4a cKO* mice was significantly lower than that of wild-type mice in the early postnatal period. The body weight of homozygous Kdm4a cKO mice was significantly lower than that of WT mice from P5, and the body weight of heterozygous Kdm4a cKO mice was significantly lower than WT mice from P13. *Kdm4a*$^{f/f}$, n = 6; *Nes-Cre;Kdm4a*$^{f/w}$, n = 5; *Nes-Cre;Kdm4a*$^{f/f}$, n = 5. Two-way ANOVA, $F_{2,78}$ = 44.67, p < 0.0001. * p < 0.05, ** p < 0.01, *** p < 0.001, and **** p < 0.0001. Data are presented as mean ± s.e.m.

Supplementary Figure 15 Uncropped western blots

Fig. 1h

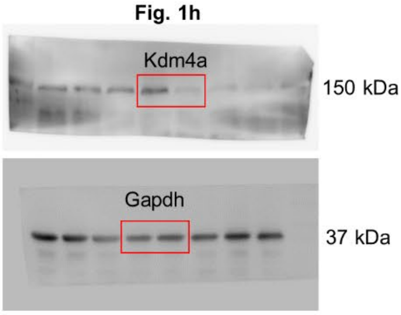

Fig. 3c

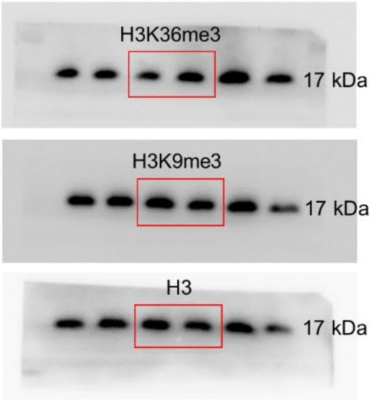

Fig. 3g

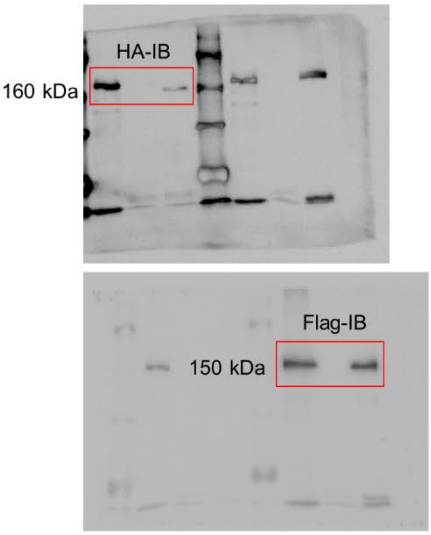

Fig. 6a

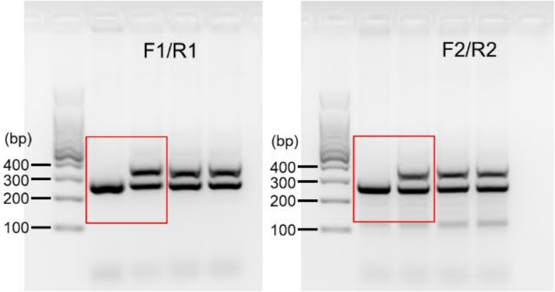

Fig. 6d

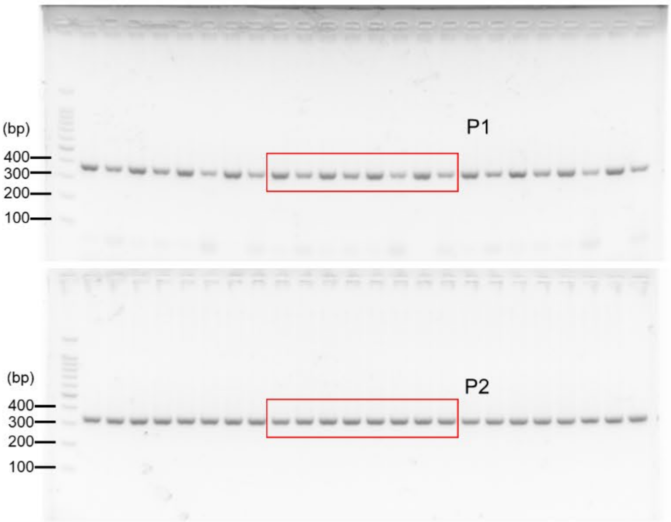

Fig. 6c

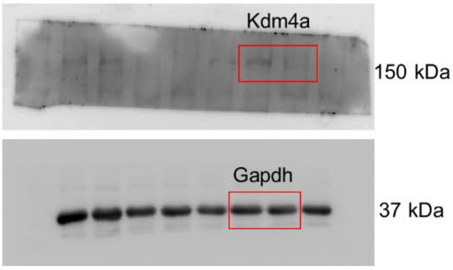

Supplementary Figure 16 Uncropped western blots

Supplementary Fig. 1c

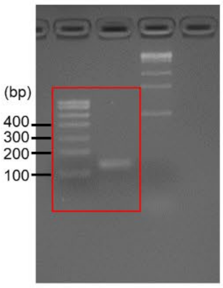

Supplementary Fig. 1d

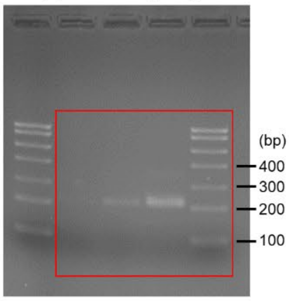

Supplementary Fig. 1n

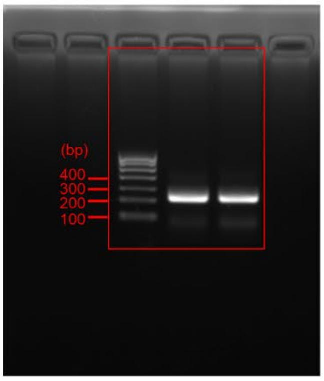

Supplementary Fig. 12d

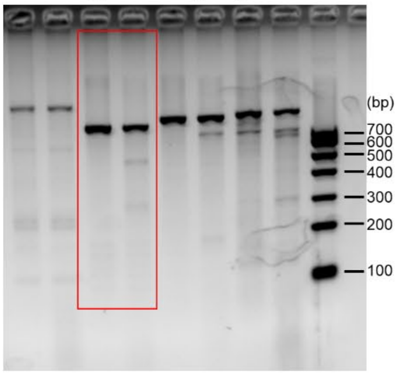

Supplementary Fig. 5b

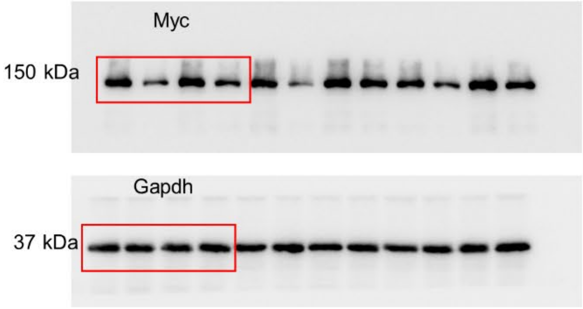

Supplementary Fig. 9c

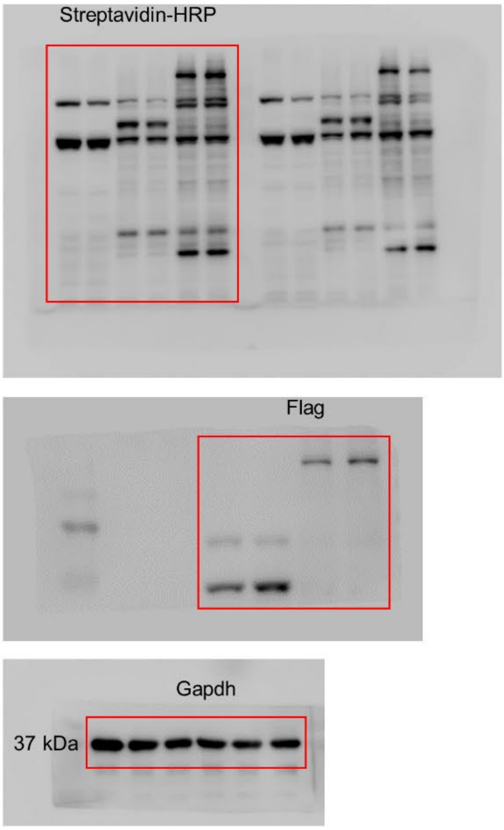

Supplementary Fig. 10b

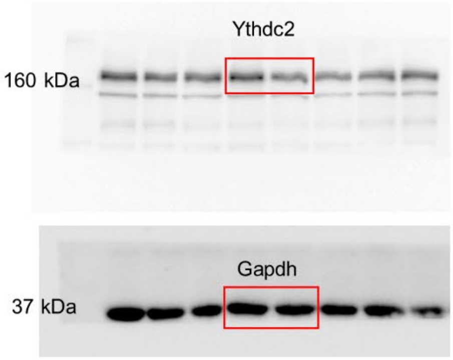

Supplement: Supplementary file 1 — Supplementary Information [file 41467_2024_50218_MOESM1_ESM.pdf]
