# Supplementary material for: Kdm4a is an activity downregulated barrier to generate engrams for memory separation
Source: Nat Commun. 2024 Jul 13;15:5887. doi: 10.1038/s41467-024-50218-y (PMC11246488; doi:10.1038/s41467-024-50218-y)
Supplement: Supplementary file 3 — Reporting Summary [file 41467_2024_50218_MOESM3_ESM.pdf]

nature portfolio

Corresponding author(s): Hong Xie and Ji-Song Guan

Last updated by author(s): Jun 8, 2024

Reporting Summary

Nature Portfolio wishes to improve the reproducibility of the work that we publish. This form provides structure for consistency and transparency in reporting. For further information on Nature Portfolio policies, see our Editorial Policies and the Editorial Policy Checklist.

Statistics

For all statistical analyses, confirm that the following items are present in the figure legend, table legend, main text, or Methods section.

n/a	Confirmed	
☐	☒	The exact sample size (*n*) for each experimental group/condition, given as a discrete number and unit of measurement
☐	☒	A statement on whether measurements were taken from distinct samples or whether the same sample was measured repeatedly
☐	☒	The statistical test(s) used AND whether they are one- or two-sided<br>*Only common tests should be described solely by name; describe more complex techniques in the Methods section.*
☒	☐	A description of all covariates tested
☐	☒	A description of any assumptions or corrections, such as tests of normality and adjustment for multiple comparisons
☐	☒	A full description of the statistical parameters including central tendency (e.g. means) or other basic estimates (e.g. regression coefficient) AND variation (e.g. standard deviation) or associated estimates of uncertainty (e.g. confidence intervals)
☐	☒	For null hypothesis testing, the test statistic (e.g. *F*, *t*, *r*) with confidence intervals, effect sizes, degrees of freedom and *P* value noted<br>*Give P values as exact values whenever suitable.*
☒	☐	For Bayesian analysis, information on the choice of priors and Markov chain Monte Carlo settings
☒	☐	For hierarchical and complex designs, identification of the appropriate level for tests and full reporting of outcomes
☒	☐	Estimates of effect sizes (e.g. Cohen's *d*, Pearson's *r*), indicating how they were calculated

Our web collection on statistics for biologists contains articles on many of the points above.

Software and code

Policy information about availability of computer code

Data collection	Cells were sorted with the flow cytometer BD FACS Aria III.<br>RNA Sequencing data was generated by Illumina HiSeq 2500 platform.<br>Real-time qPCR data was generated by QuantStudio 7 Flex System.<br>Confocal images were acquired using Nikon CSU-W1 SoRa Microscope.
Data analysis	GraphPad Prism version 9.0.0<br>ImageJ Fiji version 1.53q<br>Icy version 2.4.3.0<br>FlowJo 10.6.2<br>BD FACSDiva 8.0.1<br>Quality control of plasmid sgRNA library was performed using custom MATLAB codes. All the other sequencing data were analyzed using published protocols and codes described in the Methods sections. The custom codes are available on Zendo: https://zenodo.org/records/11523126.

For manuscripts utilizing custom algorithms or software that are central to the research but not yet described in published literature, software must be made available to editors and reviewers. We strongly encourage code deposition in a community repository (e.g. GitHub). See the Nature Portfolio guidelines for submitting code & software for further information.

Data

Policy information about availability of data

All manuscripts must include a data availability statement. This statement should provide the following information, where applicable:

- Accession codes, unique identifiers, or web links for publicly available datasets
- A description of any restrictions on data availability
- For clinical datasets or third party data, please ensure that the statement adheres to our policy

Raw data are available in the Source Data and the Supplementary files .The Supplementary Tables are available on figshare: https://doi.org/10.6084/m9.figshare.25990495. Sequencing data associated with this study have been deposited at the NCBI Gene Expression Omnibus under accession number GSE269325. In vivo neuronal activity downregulated gene lists were downloaded from GSE77067 (novelty exploration), GSE82013 (electroconvulsive stimulation) and GSE125068 (kainic acid).

Research involving human participants, their data, or biological material

Policy information about studies with human participants or human data. See also policy information about sex, gender (identity/presentation), and sexual orientation and race, ethnicity and racism.

Reporting on sex and gender	N/A
Reporting on race, ethnicity, or other socially relevant groupings	N/A
Population characteristics	N/A
Recruitment	N/A
Ethics oversight	N/A

Note that full information on the approval of the study protocol must also be provided in the manuscript.

Field-specific reporting

Please select the one below that is the best fit for your research. If you are not sure, read the appropriate sections before making your selection.

☒ Life sciences ☐ Behavioural & social sciences ☐ Ecological, evolutionary & environmental sciences

For a reference copy of the document with all sections, see nature.com/documents/nr-reporting-summary-flat.pdf

Life sciences study design

All studies must disclose on these points even when the disclosure is negative.

Sample size	No statistical method was used to predetermine the sample size. The number of samples in each experiment was chosen based on standard practice in the field. All sample size was outlined in the legend of each figure, our Source Data and the Methods section. Animals, both male and female, (2-6 months old) were randomly assigned to the experimental groups. For animal behavioral tests, 6-13 mice per group were used. For immuno-staining experiments, 3 biological replicates were used. For RNA-seq, RT-qPCR and WB, 3-4 biological replicates per group were used.
Data exclusions	Data was not excluded from analysis.
Replication	All attempts of replication were successful and described in the figure legend, source data and Methods section.
Randomization	Samples (cells, tissues, mice) were not allocated randomly to experiments but allocated based on the treatment or genotype.
Blinding	Full blinding was not applied because samples needed to be controlled by genotype or treatment.

Behavioural & social sciences study design

All studies must disclose on these points even when the disclosure is negative.

Study description	*Briefly describe the study type including whether data are quantitative, qualitative, or mixed-methods (e.g. qualitative cross-sectional, quantitative experimental, mixed-methods case study).*

Research sample

State the research sample (e.g. Harvard university undergraduates, villagers in rural India) and provide relevant demographic information (e.g. age, sex) and indicate whether the sample is representative. Provide a rationale for the study sample chosen. For studies involving existing datasets, please describe the dataset and source.

Sampling strategy

Describe the sampling procedure (e.g. random, snowball, stratified, convenience). Describe the statistical methods that were used to predetermine sample size OR if no sample-size calculation was performed, describe how sample sizes were chosen and provide a rationale for why these sample sizes are sufficient. For qualitative data, please indicate whether data saturation was considered, and what criteria were used to decide that no further sampling was needed.

Data collection

Provide details about the data collection procedure, including the instruments or devices used to record the data (e.g. pen and paper, computer, eye tracker, video or audio equipment) whether anyone was present besides the participant(s) and the researcher, and whether the researcher was blind to experimental condition and/or the study hypothesis during data collection.

Timing

Indicate the start and stop dates of data collection. If there is a gap between collection periods, state the dates for each sample cohort.

Data exclusions

If no data were excluded from the analyses, state so OR if data were excluded, provide the exact number of exclusions and the rationale behind them, indicating whether exclusion criteria were pre-established.

Non-participation

State how many participants dropped out/declined participation and the reason(s) given OR provide response rate OR state that no participants dropped out/declined participation.

Randomization

If participants were not allocated into experimental groups, state so OR describe how participants were allocated to groups, and if allocation was not random, describe how covariates were controlled.

Ecological, evolutionary & environmental sciences study design

All studies must disclose on these points even when the disclosure is negative.

Study description

Briefly describe the study. For quantitative data include treatment factors and interactions, design structure (e.g. factorial, nested, hierarchical), nature and number of experimental units and replicates.

Research sample

Describe the research sample (e.g. a group of tagged Passer domesticus, all Stenocereus thurberi within Organ Pipe Cactus National Monument), and provide a rationale for the sample choice. When relevant, describe the organism taxa, source, sex, age range and any manipulations. State what population the sample is meant to represent when applicable. For studies involving existing datasets, describe the data and its source.

Sampling strategy

Note the sampling procedure. Describe the statistical methods that were used to predetermine sample size OR if no sample-size calculation was performed, describe how sample sizes were chosen and provide a rationale for why these sample sizes are sufficient.

Data collection

Describe the data collection procedure, including who recorded the data and how.

Timing and spatial scale

Indicate the start and stop dates of data collection, noting the frequency and periodicity of sampling and providing a rationale for these choices. If there is a gap between collection periods, state the dates for each sample cohort. Specify the spatial scale from which the data are taken

Data exclusions

If no data were excluded from the analyses, state so OR if data were excluded, describe the exclusions and the rationale behind them, indicating whether exclusion criteria were pre-established.

Reproducibility

Describe the measures taken to verify the reproducibility of experimental findings. For each experiment, note whether any attempts to repeat the experiment failed OR state that all attempts to repeat the experiment were successful.

Randomization

Describe how samples/organisms/participants were allocated into groups. If allocation was not random, describe how covariates were controlled. If this is not relevant to your study, explain why.

Blinding

Describe the extent of blinding used during data acquisition and analysis. If blinding was not possible, describe why OR explain why blinding was not relevant to your study.

Did the study involve field work? ☐ Yes ☐ No

Field work, collection and transport

Field conditions

Describe the study conditions for field work, providing relevant parameters (e.g. temperature, rainfall).

Location

State the location of the sampling or experiment, providing relevant parameters (e.g. latitude and longitude, elevation, water depth).

Access & import/export

Describe the efforts you have made to access habitats and to collect and import/export your samples in a responsible manner and in

Access & import/export	compliance with local, national and international laws, noting any permits that were obtained (give the name of the issuing authority, the date of issue, and any identifying information).
Disturbance	Describe any disturbance caused by the study and how it was minimized.

Reporting for specific materials, systems and methods

We require information from authors about some types of materials, experimental systems and methods used in many studies. Here, indicate whether each material, system or method listed is relevant to your study. If you are not sure if a list item applies to your research, read the appropriate section before selecting a response.

Materials & experimental systems

n/a	Involved in the study	
☐	☒	Antibodies
☐	☒	Eukaryotic cell lines
☒	☐	Palaeontology and archaeology
☐	☒	Animals and other organisms
☒	☐	Clinical data
☒	☐	Dual use research of concern
☒	☐	Plants

Methods

n/a	Involved in the study	
☒	☐	ChIP-seq
☐	☒	Flow cytometry
☒	☐	MRI-based neuroimaging

Antibodies

Antibodies used

Primary antibodies:
Mouse anti-GFP (ABclonal AE012) 1:100
Rabbit antiGFP (Thermo A11122) 1:1000
Rabbit anti-mCherry (Abcam ab167453) 1:1000
Mouse anti-Flag-tag (Yeasen 30503ES60) 1:1000
Rabbit anti-HA-tag (Beyotime AF0039) 1:1000
Mouse anti-BrdU (Sigma B2531-.2ML) NRO 2 μg
Rabbit anti-GAPDH (Cell Signaling Technology 2118S) 1:1000
Goat anti-Histone H3 (Santa Cruz sc-8654) 1:1000
Rabbit anti-H3K9me3 (Abcam ab8898) 1:1000, ChIP 2 μg
Rabbit anti-H3K36me3 (Abcam ab9050) 1:1000, ChIP 2 μg
Rabbit anti-m6A (Beyotime AF7407) RIP 5 μg
Rabbit anti-Kdm4a (Cell Signaling Technology 5328S) 1:1000
Mouse anti-Trpm7 (Thermo MA527620) 1:500
Rabbit anti-Ythdc2 (Abcam ab220160) IB 1:1000, RIP 4 μg
Rabbit anti-NeuN (Cell Signaling Technology 24307S) 1:1000
Mouse anti-NeuN (Proteintech 66836-1-Ig) FACS 1:400
Rabbit anti-Fos (Cell Signaling Technology 2250S) 1:1000, FACS 1:1000
Rabbit anti-Egr1 (Cell Signaling Technology 4153S) 1:1000
Mouse anti-Fos (Abcam ab208942-100ul) 1:500
Mouse anti-GFAP (Cell Signaling Technology 3670T) 1:1000
Rabbit anti-Olig2 (Abcam ab109186) 1:100
Goat anti-Iba1 (Abcam ab5076) 1:500
Rabbit anti-Somatostatin (Peninsula Laboratories T-4103) 1:200
Rabbit anti-Parvalbumin (Cell Signaling Technology 80561T) 1:1000

Secondary antibodies:
For Immunostaining
Goat anti-Mouse IgG (H+L) Secondary Antibody, Alexa Fluor 555 (Thermo A21422) 1:1000
Alexa Fluor 488 AffiniPure Goat Anti-Rabbit IgG (H+L) (Yeasen 33106ES60) 1:500
Alexa Fluor 647- AffiniPure Goat Anti-Mouse IgG (H+L) (Yeasen 33213ES60) 1:500
Alexa Fluor 647-conjugated Streptavidin (Yeasen 35104ES60) 1:500
Alexa Fluor 647 AffiniPure Rabbit Anti-Goat IgG (H+L) (Yeasen 33713ES60) 1:500
For Western Blot
Goat anti-Rabbit IgG (H+L) HRP (BioWorld BS13278) 1:10000
Goat anti-Mouse IgG (H+L) HRP (SparkJade EF0001) 1:10000
Donkey anti-Goat IgG (H+L) HRP (bioss bs-0294D-HRP-100ul) 1:10000

Validation

All antibodies are commercially available and have been validated by the manufactures.
The Mouse anti-GFP (ABclonal AE012) were previously validated in mouse for IF/WB in their website (https://abclonal.com.cn/catalog/AE012). Also validated in Xin Yang et al. Cell Research. 2017. It has been cited by 191 publications.
The Rabbit antiGFP (Thermo A11122) were previously validated in mouse for IF in their website (https://www.thermofisher.cn/cn/zh/antibody/product/GFP-Antibody-Polyclonal/A-11122). Also validated in Bolderson E et al. Nucleic acids research. 2017. It has been cited by 1783 publications.
The Rabbit anti-mCherry (Abcam ab167453) were previously validated in mouse for IF in their website (https://www.abcam.cn/products/primary-antibodies/mcherry-antibody-ab167453.html). Also validated in Chen ZS et al. Nat Commun 14:8420 (2023). It has

been cited by 393 publications.
The Mouse anti-Flag-tag (Yeasen 30503ES60) were previously validated in mouse for IP in their website (https://www.yeasen.com/products/detail/89). Also validated in Dong W et al. Nature. 2021 ;589(7843):586-590. It has been cited by 9 publications.
The Rabbit anti-HA-tag (Beyotime AF0039) were previously validated in mouse for IP in their website (https://m.beyotime.com/mobilegoods.do?method=code&code=AF0039). Also validated in Ruirui Jia et al. Nucleic Acids Res. 2021. It has been cited by 10 publications.
The Mouse anti-BrdU (Sigma B2531-.2ML) were previously validated in mouse for IP in their website (https://www.sigmaaldrich.cn/CN/en/product/sigma/b8434). Also validated in Yan Cheng et al. Cell reports, 2020. It has been cited by 178 publications.
The Rabbit anti-GAPDH (Cell Signaling Technology 2118S) were previously validated in mouse for WB in their website (https://www.cellsignal.cn/products/primary-antibodies/gapdh-14c10-rabbit-mab/2118). Also validated in Xiling Bian, et. al. Elife, 2023. It has been cited by 7799 publications.
The Goat anti-Histone H3 (Santa Cruz sc-8654) were previously validated in mouse for WB in their website (https://www.scbt.com/p/histone-h3-antibody-c-16). Also validated in Lončarić, M. et al. 2023. Cellular & molecular biology letters. It has been cited by 48 publications.
The Rabbit anti-H3K9me3 (Abcam ab8898) were previously validated in mouse for WB/ChIP in their website (https://www.abcam.com/products/primary-antibodies/histone-h3-tri-methyl-k9-antibody-chip-grade-ab8898.html). Also validated in Meers MP et al. Nat Biotechnol 41:708-716 (2023). It has been cited by 1603 publications.
The Rabbit anti-H3K36me3 (Abcam ab9050) were previously validated in mouse for WB/ChIP in their website (https://www.abcam.cn/products/primary-antibodies/histone-h3-tri-methyl-k36-antibody-chip-grade-ab9050.html). Also validated in Stewart-Morgan KR et al. Nat Cell Biol 25:183-193 (2023). It has been cited by 904 publications.
The Rabbit anti-m6A (Beyotime AF7407) were previously validated in mouse for IP in their website (https://www.beyotime.com/product/AF7407.htm).
The Rabbit anti-Kdm4a (Cell Signaling Technology 5328S) were previously validated in mouse for WB/IF in their website (https://www.cellsignal.com/products/primary-antibodies/jmjd2a-c37e5-rabbit-mab/5328). Also validated in Chloe-Anne Martinez, et. al. J Biol Chem. 2022. It has been cited by 24 publications.
The Mouse anti-Trpm7 (Thermo MA527620) were previously validated in mouse for IF in their website (https://www.thermofisher.cn/antibody/primary/target/trpm7). Also validated in Zhi-Guo Zou et al. Clin Sci (Lond). 2020. It has been cited by 3 publications.
The Rabbit anti-Ythdc2 (Abcam ab220160) were previously validated in mouse for WB/RIP in their website (https://www.abcam.cn/products/primary-antibodies/ythdc2-antibody-epr21820-49-ab220160.html). Also validated in Jiao Y et al. Acta Diabetol 60:387-399 (2023). It has been cited by 10 publications.
The Rabbit anti-NeuN (Cell Signaling Technology 24307S) were previously validated in mouse for IF in their website (https://www.cellsignal.com/products/primary-antibodies/neun-d4g4o-xp-rabbit-mab/24307). Also validated in Guixiang Yang, et. Al. Gene Ther. 2023. It has been cited by 192 publications.
The Mouse anti-NeuN (Proteintech 66836-1-Ig) were previously validated in mouse for FACS in their website (https://www.ptgcn.com/Products/NeuN-Antibody-66836-1-Ig.htm). Also validated in Shahzad S Khan et al. Elife. 2021. It has been cited by 69 publications.
The Rabbit anti-Fos (Cell Signaling Technology 2250S) were previously validated in mouse for IF/FACS in their website (https://www.cellsignal.com/products/primary-antibodies/c-fos-9f6-rabbit-mab/2250). Also validated in Li Ma, et al. Nat Commun. 2023. It has been cited by 844 publications.
The Rabbit anti-Egr1 (Cell Signaling Technology 4153S) were previously validated in mouse for IF in their website (https://www.cellsignal.com/products/primary-antibodies/egr1-15f7-rabbit-mab/4153). Also validated in Zunxian Wang, et. al. Cancer Biol Ther. 2023. It has been cited by 167 publications.
The Mouse anti-GFAP (Cell Signaling Technology 3670T) were previously validated in mouse for IF in their website (https://www.cellsignal.cn/products/primary-antibodies/gfap-ga5-mouse-mab/3670). Also validated in Yanran Bi, et. al. Genes Dis. 2024. It has been cited by 764 publications.
The Rabbit anti-Olig2 (Abcam ab109186) were previously validated in mouse for IF in their website (https://www.abcam.cn/products/primary-antibodies/olig2-antibody-epr2673-ab109186.html). Also validated in Peng K et al. CNS Neurosci Ther 28:842-850 (2022). It has been cited by 132 publications.
The Goat anti-Iba1 (Abcam ab5076) were previously validated in mouse for IF in their website (https://www.abcam.cn/products/primary-antibodies/iba1-antibody-ab5076.html). Also validated in McNamara NB et al. Nature 613:120-129 (2023). It has been cited by 1145 publications.
The Rabbit anti-Somatostatin (Peninsula Laboratories T-4103) were previously validated in mouse for IF in their website (https://www.bma.ch/antibodies/p/t-4103).
The Rabbit anti-Parvalbumin (Cell Signaling Technology 80561T) were previously validated in mouse for IF in their website (https://www.cellsignal.cn/products/primary-antibodies/parvalbumin-e8n2u-xp-rabbit-mab/80561). Also validated in Yunhu Bai, et. al. Nat Commun. 2023. It has been cited by 2 publications.

Eukaryotic cell lines

Policy information about cell lines and Sex and Gender in Research

Cell line source(s)	HEK293T (ATCC CRL-3216)<br>HEK293FT (ATCC CRL-3249)<br>Neuro-2a (ATCC CCL-131)<br>U2-OS (ATCC HTB-96)
Authentication	None of the cell lines have been authenticated.
Mycoplasma contamination	Cell lines were not tested for mycoplasma but no indication of contamination was observed.
Commonly misidentified lines<br>(See ICLAC register)	No misidentified cell lines were used in this study.

Palaeontology and Archaeology

Specimen provenance	*Provide provenance information for specimens and describe permits that were obtained for the work (including the name of the issuing authority, the date of issue, and any identifying information). Permits should encompass collection and, where applicable, export.*
Specimen deposition	*Indicate where the specimens have been deposited to permit free access by other researchers.*
Dating methods	*If new dates are provided, describe how they were obtained (e.g. collection, storage, sample pretreatment and measurement), where they were obtained (i.e. lab name), the calibration program and the protocol for quality assurance OR state that no new dates are provided.*

☐ Tick this box to confirm that the raw and calibrated dates are available in the paper or in Supplementary Information.

Ethics oversight	*Identify the organization(s) that approved or provided guidance on the study protocol, OR state that no ethical approval or guidance was required and explain why not.*

Note that full information on the approval of the study protocol must also be provided in the manuscript.

Animals and other research organisms

Policy information about studies involving animals; ARRIVE guidelines recommended for reporting animal research, and Sex and Gender in Research

Laboratory animals	The mice were bred from C57BL/6J background and group-housed (3-5 mice per cage) on a 12h/12h light/dark cycle with diet and water freely available. The ambient temperature was maintained at 23 ?, and humidity levels at 50%. Tg(Egr1-EGFP)GO90Gsat (GENSAT, strain #: 4847022) knockin mice, both male and female, aged 8-12 weeks, were bilaterally injected with LV-CRISPR library into the dorsal dentate gyrus (±1.5, -2.0, -2.0). Mice were allowed to recover from surgery for 2-3 weeks before all behavioral tasks. B6.Cg-Tg(Nes-cre)1Kln/J (The Jax lab, strain #: 003771) mice were crossed to Kdm4aflox/flox mice (customed from GemPharmatech, Strain #: T052202) to generate neuron-specific Kdm4a knockout mice (Nes-Cre+/Tg;Kdm4af/f). Animals, both male and female, were randomly assigned to the experimental groups.
Wild animals	This study did not involve wild animals.
Reporting on sex	N/A
Field-collected samples	This study did not include samples collected from the field.
Ethics oversight	Animals were raised on the Model Animal Platform at ShanghaiTech University. All animal protocols were approved by the Institutional Animal Care and Use Committee of the ShanghaiTech University.

Note that full information on the approval of the study protocol must also be provided in the manuscript.

Clinical data

Policy information about clinical studies

All manuscripts should comply with the ICMJE guidelines for publication of clinical research and a completed CONSORT checklist must be included with all submissions.

Clinical trial registration	*Provide the trial registration number from ClinicalTrials.gov or an equivalent agency.*
Study protocol	*Note where the full trial protocol can be accessed OR if not available, explain why.*
Data collection	*Describe the settings and locales of data collection, noting the time periods of recruitment and data collection.*
Outcomes	*Describe how you pre-defined primary and secondary outcome measures and how you assessed these measures.*

Dual use research of concern

Policy information about dual use research of concern

Hazards

Could the accidental, deliberate or reckless misuse of agents or technologies generated in the work, or the application of information presented in the manuscript, pose a threat to:

No	Yes	
☐	☐	Public health
☐	☐	National security
☐	☐	Crops and/or livestock
☐	☐	Ecosystems
☐	☐	Any other significant area

Experiments of concern

Does the work involve any of these experiments of concern:

No	Yes	
☐	☐	Demonstrate how to render a vaccine ineffective
☐	☐	Confer resistance to therapeutically useful antibiotics or antiviral agents
☐	☐	Enhance the virulence of a pathogen or render a nonpathogen virulent
☐	☐	Increase transmissibility of a pathogen
☐	☐	Alter the host range of a pathogen
☐	☐	Enable evasion of diagnostic/detection modalities
☐	☐	Enable the weaponization of a biological agent or toxin
☐	☐	Any other potentially harmful combination of experiments and agents

Plants

Seed stocks	*Report on the source of all seed stocks or other plant material used. If applicable, state the seed stock centre and catalogue number. If plant specimens were collected from the field, describe the collection location, date and sampling procedures.*
Novel plant genotypes	*Describe the methods by which all novel plant genotypes were produced. This includes those generated by transgenic approaches, gene editing, chemical/radiation-based mutagenesis and hybridization. For transgenic lines, describe the transformation method, the number of independent lines analyzed and the generation upon which experiments were performed. For gene-edited lines, describe the editor used, the endogenous sequence targeted for editing, the targeting guide RNA sequence (if applicable) and how the editor was applied.*
Authentication	*Describe any authentication procedures for each seed stock used or novel genotype generated. Describe any experiments used to assess the effect of a mutation and, where applicable, how potential secondary effects (e.g. second site T-DNA insertions, mosiacism, off-target gene editing) were examined.*

ChIP-seq

Data deposition

☐ Confirm that both raw and final processed data have been deposited in a public database such as GEO.

☐ Confirm that you have deposited or provided access to graph files (e.g. BED files) for the called peaks.

Data access links<br>*May remain private before publication.*	*For "Initial submission" or "Revised version" documents, provide reviewer access links. For your "Final submission" document, provide a link to the deposited data.*
Files in database submission	*Provide a list of all files available in the database submission.*
Genome browser session<br>(e.g. UCSC)	*Provide a link to an anonymized genome browser session for "Initial submission" and "Revised version" documents only, to enable peer review. Write "no longer applicable" for "Final submission" documents.*

Methodology

Replicates	*Describe the experimental replicates, specifying number, type and replicate agreement.*
Sequencing depth	*Describe the sequencing depth for each experiment, providing the total number of reads, uniquely mapped reads, length of reads and whether they were paired- or single-end.*
Antibodies	*Describe the antibodies used for the ChIP-seq experiments; as applicable, provide supplier name, catalog number, clone name, and lot number.*
Peak calling parameters	*Specify the command line program and parameters used for read mapping and peak calling, including the ChIP, control and index files used.*

Data quality	*Describe the methods used to ensure data quality in full detail, including how many peaks are at FDR 5% and above 5-fold enrichment.*
Software	*Describe the software used to collect and analyze the ChIP-seq data. For custom code that has been deposited into a community repository, provide accession details.*

Flow Cytometry

Plots

Confirm that:

☒ The axis labels state the marker and fluorochrome used (e.g. CD4-FITC).

☒ The axis scales are clearly visible. Include numbers along axes only for bottom left plot of group (a 'group' is an analysis of identical markers).

☒ All plots are contour plots with outliers or pseudocolor plots.

☒ A numerical value for number of cells or percentage (with statistics) is provided.

Methodology

Sample preparation	Mouse was perfused with chilled perfusion buffer (115 mM Choline chloride, 2.5 mM KCl, 1.25 mM NaH2PO4, 26 mM NaHCO3, 10 mM glucose, 8 mM MgSO4, 1 mM Sodium L-ascorbate and 3 mM Sodium pyruvate, pH 7.4) . Mouse brain was dissected immediately and immersed in the ice-cold modified EBSS buffer (116 mM NaCl, 5.4 mM KCl, 1 mM NaH2PO4, 26 mM NaHCO3, 1.5 mM CaCl2, 1 mM MgSO4, 0.5 mM EDTA, 25 mM glucose and 1 mM L-Cysteine, pH 7.4). The dentate gyrus (DG) was microdissected and collected for either papain-based enzymatic dissociation or Dounce homogenization. The isolated EGFP/mCherry-expressing neurons or immunostained nuclei were filtered with 100/40-µm cell strainer and used for FACS.
Instrument	Flow cytometric data was collected on a BD Aria III sorter.
Software	Data was collected on BD FACSDiva 8.0.1 software, and analyzed on FlowJo 10.6.2 software.
Cell population abundance	Cell numbers were checked by hemocytometer manually. EGFP/mCherry and immunostained signals were quantified under fluorescence microscope.
Gating strategy	FSC/SSC were used for initial sorting. DAPI were used for excluding the dead cells. Hoechst-33342 were used to identify the nuclei. GFP/mCherry were used for sorting.

☒ Tick this box to confirm that a figure exemplifying the gating strategy is provided in the Supplementary Information.

Magnetic resonance imaging

Experimental design

Design type	*Indicate task or resting state; event-related or block design.*
Design specifications	*Specify the number of blocks, trials or experimental units per session and/or subject, and specify the length of each trial or block (if trials are blocked) and interval between trials.*
Behavioral performance measures	*State number and/or type of variables recorded (e.g. correct button press, response time) and what statistics were used to establish that the subjects were performing the task as expected (e.g. mean, range, and/or standard deviation across subjects).*

Acquisition

Imaging type(s)	*Specify: functional, structural, diffusion, perfusion.*
Field strength	*Specify in Tesla*
Sequence & imaging parameters	*Specify the pulse sequence type (gradient echo, spin echo, etc.), imaging type (EPI, spiral, etc.), field of view, matrix size, slice thickness, orientation and TE/TR/flip angle.*
Area of acquisition	*State whether a whole brain scan was used OR define the area of acquisition, describing how the region was determined.*

Diffusion MRI ☐ Used ☐ Not used

Preprocessing

Preprocessing software	*Provide detail on software version and revision number and on specific parameters (model/functions, brain extraction, segmentation, smoothing kernel size, etc.).*

Normalization | *If data were normalized/standardized, describe the approach(es): specify linear or non-linear and define image types used for transformation OR indicate that data were not normalized and explain rationale for lack of normalization.*

Normalization template | *Describe the template used for normalization/transformation, specifying subject space or group standardized space (e.g. original Talairach, MNI305, ICBM152) OR indicate that the data were not normalized.*

Noise and artifact removal | *Describe your procedure(s) for artifact and structured noise removal, specifying motion parameters, tissue signals and physiological signals (heart rate, respiration).*

Volume censoring | *Define your software and/or method and criteria for volume censoring, and state the extent of such censoring.*

Statistical modeling & inference

Model type and settings | *Specify type (mass univariate, multivariate, RSA, predictive, etc.) and describe essential details of the model at the first and second levels (e.g. fixed, random or mixed effects; drift or auto-correlation).*

Effect(s) tested | *Define precise effect in terms of the task or stimulus conditions instead of psychological concepts and indicate whether ANOVA or factorial designs were used.*

Specify type of analysis: ☐ Whole brain ☐ ROI-based ☐ Both

Statistic type for inference | *Specify voxel-wise or cluster-wise and report all relevant parameters for cluster-wise methods.*

(See Eklund et al. 2016)

Correction | *Describe the type of correction and how it is obtained for multiple comparisons (e.g. FWE, FDR, permutation or Monte Carlo).*

Models & analysis

n/a	Involved in the study
☐	☐ Functional and/or effective connectivity
☐	☐ Graph analysis
☐	☐ Multivariate modeling or predictive analysis

Functional and/or effective connectivity | *Report the measures of dependence used and the model details (e.g. Pearson correlation, partial correlation, mutual information).*

Graph analysis | *Report the dependent variable and connectivity measure, specifying weighted graph or binarized graph, subject- or group-level, and the global and/or node summaries used (e.g. clustering coefficient, efficiency, etc.).*

Multivariate modeling and predictive analysis | *Specify independent variables, features extraction and dimension reduction, model, training and evaluation metrics.*
